# PARK2 Suppresses Proliferation and Tumorigenicity in Non-small Cell Lung Cancer

**DOI:** 10.3389/fonc.2019.00790

**Published:** 2019-08-23

**Authors:** Huijie Duan, Zhong Lei, Fei Xu, Tao Pan, Demin Lu, Peili Ding, Chunpeng Zhu, Chi Pan, Suzhan Zhang

**Affiliations:** ^1^Department of Medical Oncology, The Second Affiliated Hospital, Zhejiang University School of Medicine, Hangzhou, China; ^2^Key Laboratory of Cancer Prevention and Intervention, China National Ministry of Education, The Second Affiliated Hospital, School of Medicine, Cancer Institute, Zhejiang University, Hangzhou, China; ^3^Department of Orthopedics Research Institute, The Second Affiliated Hospital, School of Medicine, Zhejiang University, Hangzhou, China; ^4^Department of Oncology, Municipal Hospital of Qingdao, Qingdao, China; ^5^Department of Breast Surgery, The Second Affiliated Hospital, School of Medicine, Zhejiang University, Hangzhou, China; ^6^Department of Gastroenterology, The Second Affiliated Hospital, Zhejiang University School of Medicine, Hangzhou, China; ^7^Research Center for Air Pollution and Health, School of Medicine, Zhejiang University, Hangzhou, China

**Keywords:** *PARK2*, non-small cell lung cancer, proliferation, metastasis, EGFR/AKT/mTOR

## Abstract

**Aims:** *PARK2* mutation is originally associated with the progression of Parkinson's disease. In recent years, *PARK2* has been reported as a tumor suppressor gene in various cancers, including lung cancer. However, the biological functions and potential molecular mechanisms of PARK2 in non-small cell lung cancer (NSCLC) are still unclear.

**Methods:** The level of PARK2 expression in 32 tissue samples of NSCLC and matched non-tumor lung tissues was detected by Western blot, and 64 specimens of NSCLC tissues were detected by immunohistochemistry. H1299 and H460 cell lines were used to *PARK2* overexpression models, and H460 cell line was also used to *PARK2* knockdown model. Using cell viability, colony formation, cell cycle, apoptosis, migration, and invasion assay, the biological functions of PARK2 were evaluated and the potential molecular mechanism of PARK2 was investigated *in vitro*. Meanwhile, 22 nude mice were employed for *in vivo* studies.

**Results:** Western blot analysis revealed a decrease of PARK2 protein expression in human NSCLC samples. Immunohistochemistry also identified a vastly reduced expression of PARK2 in NSCLC (72%) and low PARK2 expression was significantly associated with tumor histological grade, lymph node metastasis and advanced TNM stage. Overexpression of *PARK2* suppressed cell proliferation, colony formation, migration, and invasion, arrested cell cycle progression in the G1 phase, and induced apoptosis in human non-small cell lines H1299 and H460 *in vitro*. Meanwhile, knockdown of *PARK2* had the opposite biological functions. In addition, PARK2 significantly decreased the tumor volumes in subcutaneous xenograft model and reduced the incidence of metastatic tumors in the transfer model. Exploration of the molecular mechanism of PARK2 in NSCLC showed that PARK2 negatively regulated the EGFR/AKT/mTOR signaling pathway.

**Conclusions:** *PARK2* was an important tumor suppressor in NSCLC, which might inhibit cancer growth and metastases through the down regulation of the EGFR/AKT/mTOR signaling pathway.

## Introduction

Lung cancer is the leading cause of cancer death globally. Approximately 1.8 million new lung cancer cases are recorded annually, and the number increases every year (1, 2). In China, lung cancer is the most commonly diagnosed type of cancer, attributable in large part to smoking and severe air pollution (3). Approximately 57% of lung cancers are diagnosed at the advanced stage because it is typically asymptomatic early. Consequently, the 5-year survival rate is only 27% for regional cases and 4% for advanced cases (4, 5). Non-small cell lung cancer (NSCLC) accounts for the largest proportion (~85%) of lung cancer (6). Fully understanding the pathogenesis and characters of lung cancer would be helpful to clinical treatment. In past decades, numerous studies have shown that lung cancer genesis and progression are associated with the mutation, deletion, inactivation, and abnormal expression of oncogenes and anti-oncogenes. Several genes related to lung cancer were identified, such as epidermal growth factor receptor (EGFR) and ALK (7, 8). Understanding the functions and underlying mechanisms of these genes is essential to the diagnosis, treatment, and prognosis of lung cancer. However, plenty of genes and their functions and mechanisms remain largely unknown in lung cancer. Thus, it is necessary to investigate the genes related to the occurrence and development of lung cancer on metabolism function and molecular level.

*PARK2* is located on the long arm of chromosome 6q25.2-q27 and was first reported for its role in the pathogenesis of autosomal recessive juvenile parkinsonism (9). *PARK2* encodes a RING-between-RING E3 ubiquitin ligase, which mediates protein degradation through the ubiquitin–proteasome system (10–14). The chromosome 6q25.2-q27 is a fragile region and prone to breakage and rearrangement, with ~500 breakpoint junctions involving *PARK2* (11). Studies have illuminated that PARK2 associated with protein turnover, stress response, mitochondria, homeostasis, xenophagy, metabolism, and many other cellular processes regulating cell growth and survival (15). Cesari et al. revealed that the loss of heterozygosity observed at chromosome 6q25–q26 leads the initiation and/or progression of cancer by inactivating or reducing the expression of *PARK2* (14). Subsequent studies also confirmed that *PARK2* was a tumor suppressor and that PARK2 deficiency or inactivation may contribute to uncontrolled cell growth and tumor formation (16). Although the precise association between *PARK2* and cancer susceptibility is not well-understood, the deletion, copy number alteration, mutations, and protein expression of PARK2 have been found in several types of cancers, such as glioblastoma, breast, ovarian, liver, colorectal, and lung (15, 17–25). Xiong et al. have identified *PARK2* mutation as a single genetic susceptibility factor for lung cancer (19) and it could become an independent prognostic marker in advanced colorectal cancer (26). Inactivation of PARK2 may play a critical role in carcinogenesis (27). However, the full extent of the role and functional mechanisms of PARK2 in NSCLC are not completely clear and highlight the need for further investigation. The aim of this study is to investigate the biological functions and molecular mechanisms of PARK2 in the tumorigenesis and development of NSCLC with *in vitro* and *in vivo* models.

## Materials and Methods

### Tissue Samples

Human NSCLC tissue samples and its adjacent non-tumor lung tissues were obtained by surgical resection from the Biobank at the Second Affiliated Hospital of Zhejiang University School of Medicine. Thirty-two pairs of specimens were stored at −80°C after surgery and another 64 pairs of specimens were paraffin-embedded tissues. The frozen and paraffin-embedded samples were not from the same donor. All tumor samples were confirmed by two different pathologists and classified according to the 7th AJCC-TNM classification of tumors. Patients were pre-operatively examined by CT and MRI scans and/or biopsy before the surgical resection. This project was approved by the Institutional Medical Ethics Committee of the Second Affiliated Hospital of Zhejiang University, and obtaining informed consent before the patients undergo surgery.

### Cell Lines and Cultivation

Human NSCLC cell lines, H1299 and H460, were obtained from the Cell Bank of the Chinese Academy of Sciences (Shanghai, China). Both cell lines were maintained in Roswell Park Memorial Institute (RPMI 1640; Gibco, USA) medium supplemented with 10% heat-inactivated fetal bovine serum (Yeasen, Shanghai, China) and 1% penicillin/streptomycin. All cells were incubated at 37°C in 5% CO_2_ incubator with a humidified atmosphere.

### Lentivirus Transfection

The overexpressed PARK2 lentivirus vector, PARK2 small hairpin RNA (shRNA) and negative control were obtained from GenePharma Corporation (Shanghai, China). The cDNA sequence of PARK2 was obtained from GenBank (NM_004562.2). The full length of PARK2 was synthesized and overexpressed using the LV5/EF-1aF/GFP/Puro vector. This artificially makes *PARK2* gene overexpression and ultimately leads to a hypernormal gene expression product by excessive transcription and translation. PARK2 shRNA (sequence: 5′-GCACCTGATCGCAACAAATAG-3′) was constructed using the LV3/pGLVH1/GFP/Puro vector and was utilized to knock down the expression of PARK2. By specifically degrading mRNA with homologous sequence of *PARK2* gene, it can prevent *PARK2* gene expression in the cell. Cells were transfected according to the manufacturer's instructions. After co-culture of the viral particles for 48 h, the medium containing 3 μg/mL puromycin was used to screen stable cell lines for a total of 10 to 14 days. Stable *PARK2* overexpressing cell lines or knockdown cell lines were confirmed by qRT-PCR, Western blot and immunofluorescence assay.

### Cell Proliferation Assay

Cells were plated at a density of 3,000 cells/well into 96-well-plates. The cell proliferation assay was assessed using the cell counting kit-8 (Yeasen, Shanghai, China) and was measured at an absorbance of 450 nm according to the manufacturer's instructions.

### Colony Formation Assay

A total of 200 cells/well were seeded into 6-well-plates, and the medium was refreshed every 2 to 3 days to observe anchorage-independent growth. After 2 weeks, colonies, which were defined as co-localized cell groups of more than 50 cells, were counted using the ImageJ analysis software.

### Wound Healing Assay

Cells were seeded into 6-well plates and grown to ~90% confluence. An artificial straight wound was scratched with a 10 μL tip through the cell monolayer. Afterward, the culture medium was replaced with serum-free culture medium. Images were obtained at 0 and 24 h (H1299) or 36 h (H460). The wound healing areas were counted using the ImageJ analysis software.

### Transwell Migration and Invasion Assays

Cell migration and invasion assays were performed using 24-well transwell plates (8 μm pore size; Costar, USA). Then, 5 × 10^4^ cells were loaded onto the upper chambers with the serum-free medium, whereas the RPMI 1640 medium plus 10% fetal bovine serum was loaded onto the lower chamber. After 16 h incubation at 37°C in 5% CO_2_ incubator, the cells migrated to the underside of the filters. Then, the cells on the upper side of the polycarbonate membrane were wiped off and stained with crystal violet. Data on the number of migrated cells are the average of the cell counts observed in 16 randomly chosen high-power fields. Only the invasion assay required the addition of 50 μL diluted Matrigel (BD Biosciences, USA) to the upper chamber of the transwell plate before the cells were loaded onto the upper chambers.

### Cell Cycle Assay

Cells were collected, washed twice with phosphate-buffered saline (PBS), and fixed with ice-cold anhydrous ethanol overnight. After subsequent rehydration in PBS for 15 min at room temperature, the samples were stained with the cell cycle staining kit (MultiSciences, Hangzhou, China) following the manufacturer's instructions and detected with a BD FACSCalibur flow cytometer (Becton Dickinson, USA).

### Cell Apoptosis Assay

The cells were seeded into 6-well-plates overnight. After serum-free starvation treatment for 48 h, the cells were collected, stained with Annexin V–APC/7-aminoactinomycin D (7-AAD) (MultiSciences, Hangzhou, China) following the manufacturer's instructions and detected with a BD FACSCalibur flow cytometer (Becton Dickinson, USA).

### Protein Extraction and Western Blot Assay

Proteins were extracted in RIPA buffer (Beyotime, Shanghai, China) containing a protease inhibitor cocktail and a phosphatase inhibitor (Bimake, USA) and determined by the BCA assay kit (Thermo, USA). After polyacrylamide gel electrophoresis, proteins were transferred to a PVDF membrane and incubated overnight at 4°C with specific primary antibodies. Antibodies against PARK2 (ab15954), CDK2 (ab32147), mTOR (ab2732), p-mTOR (ab109268), EGFR (ab52894), p-EGFR (ab40815), and AKT (ab8805) were purchased from Abcam (Cambridge, MA, USA). E-cadherin (3195), N-cadherin (13116), vimentin (5741), slug (9585) claudin-1 (13255), cyclin D1 (2978), CDK4 (12790), cyclin D3 (2936), P21 (2947), P18 (2896), cleaved caspase-3 (9664), cleaved caspase-8 (8592), cleaved caspase-9 (20750), cleaved PARP (5625), Cytochrome c (11940), p-AKT (4060), and GAPDH (2118) were obtained from Cell Signaling Technology (Boston, USA). The goat anti-rabbit IgG–HRP (HA1001-100) and goat anti-mouse IgG–HRP (HA1006) were obtained from HuaAn Biotechnology (Huabio, China). Then the PVDF membrane was incubated with secondary antibody at room temperature for 1 h. The membranes were observed with the SuperSignal West Pico Chemiluminescent Substrate (Thermo, USA). Band intensity was analyzed with Quantity One.

### Quantitative Real-Time-Polymerase Chain Reaction

Total RNA was extracted from cell lines and tissues using the TRIzol reagent (Invitrogen, Carlsbad, CA, USA) per the manufacturer's instruction. cDNA was synthesized with the PrimeScript™ RT Master Mix (TaKaRa, Japan). qRT-PCR was performed using the SYBR Premix Ex Taq™ II (TaKaRa, Japan) on a StepOnePlus Real-Time PCR system (Life Technologies, Foster, CA, USA). The relative level of gene expression was described as 2^−Δ*Ct*^ (ΔCt = Ct_PARK2_ – Ct_GAPDH_). PARK2 primers were synthesized by Sangon Biotech (Shanghai, China) and GAPDH was used as a loading control. The sequences of the primer sets were forward 5′-ATCGCAACAAATAGTCGG-3′ and reverse 5′-GGCAGGGAGTAGCCAAGT-3 for PARK2; forward 5′-GGAGCGAGATCCCTCCAAAAT-3′ and reverse 5′-GGCTGTTGTCATACTTCTCATGG-3′ for GAPDH.

### Immunohistochemistry (IHC)

Tumor tissue specimens were fixed with 4% paraformaldehyde and embedded in paraffin. Then, all sliced sections (5 μm) were de-paraffinized with xylene and ethanol. The subsequent steps were performed according to the manufacturer's instructions. Sections were incubated with primary PARK2 antibody (ab15954; Abcam), Ki-67 (ab15580; Abcam, USA) and visualized with secondary antibody (Beyotime Institute of Biotechnology, China). The percentage of positive immunostaining was scored from 0 to 4 (0, <5; 1, 5 to 25%; 2, 26 to 50%; 3, 51 to 75%; and 4, above 75%). The staining intensity was scored as follows: 0, negative; 1, weak; 2, moderate; and 3, strong. When the staining was heterogeneous, each component was scored independently (multiplying the intensity score and the area score) and received an overall assessment that translated into the indexes (–, score 0), (+, score 1~4), (++, score 5~8), and (+++, score 9~12). Each tissue section was assessed by three independent pathologists. The immunohistochemical results were categorized into negative (–) and positive expression (+~ + ++).

### Immunofluorescence

For immunofluorescence assay, the cells were fixed in 4% paraformaldehyde, incubated in 0.1% Triton-X100 and washed with PBS. The cells were stained with primary PARK2 antibody (ab15954; Abcam, USA). Afterward, the cells were counterstained with DAPI (Sigma, USA). Images were acquired by confocal laser scanning microscopy (Zeiss LSM710, Germany). Cell nuclei were stained with Hoechst 33342 (Beyotime Institute of Biotechnology, China) to observe apoptosis. Cells were processed following the manufacturer's instructions and observed at 461 nm by ultraviolet light. Images were acquired by fluorescent microscopy (Zeiss Axio Vert.A1, Germany).

### Tumor Xenograft Assay

Five-week-old female athymic mice (BALB/c^nu/nu^) were ordered from the Experimental Animal Center of Zhejiang Chinese Medical University. H1299-PARK2 and H1299-NC cell lines were harvested and injected subcutaneously into the right flank (5 × 10^6^ cells in 200 μL PBS for the subcutaneous xenograft model, six mice per group) and intravenously into the tail vein (1 × 10^6^ cells in 100 μL PBS for the metastatic model, five mice per group) of each mouse. Mice were euthanized at weeks 4 or 6. All animal procedures complied with the Zhejiang University Laws for Animal Experiments Administration and Implementation and were approved by the Zhejiang Medical University Animal Protection Committee.

### Statistics

Each experiment was performed in triplicate and independently repeated at least three times. All data are presented as the mean ± standard error of the mean. GraphPad Prism 5.0 was used to generate graphs. Statistical analyses were conducted using the SPSS 21.0 statistical software. Student's *t*-test was performed with paired comparisons. Pearson chi-square test was used for analysis between the results of IHC staining and clinical parameters. *P* < 0.05 was considered statistically significant.

## Results

### PARK2 Is Underexpressed in Human NSCLC Tissues and Cell Lines

**Figure 1:**
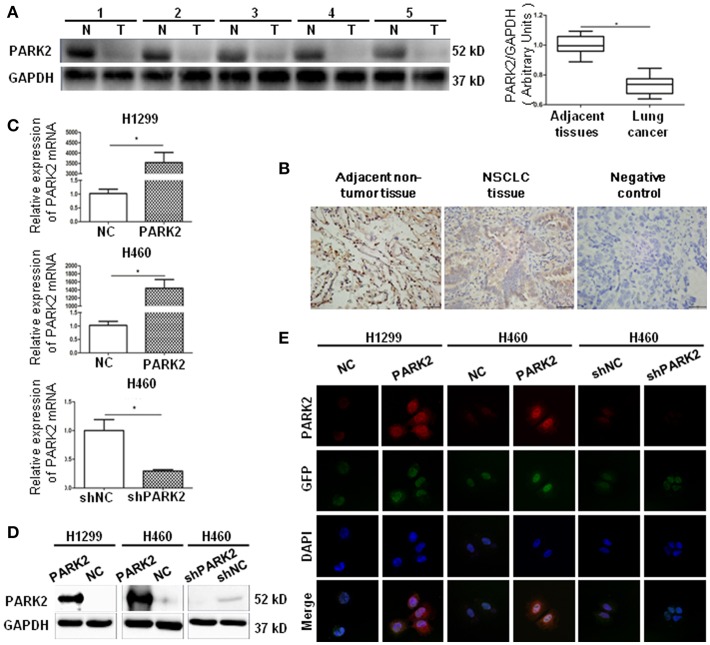
PARK2 lowly expressed in human lung cancer tissues and verified by cell transfection. **(A)** There are five representative patient samples (including four adenocarcinoma samples No. 1–3, 5; and 1 squamous cell carcinoma sample No. 4) comparing NSCLC tissues and adjacent non-tumor tissues (left legend). As detected by western blot, PARK2 protein expression was lower in human NSCLC tissues than in adjacent non-tumor tissues (right legend) (*n* = 32). **(B)** Immunohistochemistry of PARK2 expression and negative control in NSCLC tissues (middle and right) and adjacent non-tumor tissue (left) (*n* = 64, magnification × 400). **(C–E)** Overexpression of *PARK2* in H1299 and H460 cells and knockdown of *PARK2* in H460 cells as verified by qRT-PCR **(C)**, western blot **(D)**, and immunofluorescence assay (magnification × 630) **(E)** *P < 0.05.

Initially, we evaluated PARK2 expression in human NSCLC tissues and adjacent non-tumor lung tissues (*n* = 32) by Western blot assay. PARK2 was significantly decreased in NSCLC tissues vs. its adjacent non-tumor lung tissues (*P* < 0.05; Figure 1A), and the clinical characters were detailed in Table 1. Using IHC assay, we detected PARK2 in 64 paraffin-embedded NSCLC tissues with the purpose to further explore the correlations between PARK2 expression and the clinicopathologic features of NSCLC (Figure 1B). The positive rate of IHC among NSCLC tissue samples was 28% (18/64), whereas, 72% (46/64) stained negative for PARK2. Low expression of PARK2 was closely correlated to advanced TNM stage, lymph node metastasis and histological grade in NSCLC (Table 2).

**Table 1:** The clinical character of 32 NSCLC patients.

**Parameter**	**Sex**		**Age(years)**		**Histological grade**		**Histology**		**Lymph node**		**TNM stage (AJCC)**	
	**Male**	**Female**	**≤60**	**>60**	**Well and morderate**	**Poor**	**Adenocarcinoma**	**Squamous cell carcinoma (SCC)**	**N0**	**N1, N2**	**I**	**II, III**
Case	13	19	13	19	15	17	21	11	15	17	15	17

**Table 2:** Clinicopathologic features and the expression status of PARK2 in 64 NSCLC patients.

**Parameter**	**Case**	**PARK2 expression**		***P*-value**
		**Negative**	**Positive**	
Sex				
Male	39	18	11	1.000
Female	25	18	7	
Age (years)				
≤60	31	21	10	0.581
>60	33	25	8	
Histological grade				
Well	19	8	11	0.002
Moderate or poor	45	38	7	
Histology				
Adenocarcinoma	37	25	12	0.413
Squamous cell carcinoma(SCC)	27	21	6	
Tumor invasion				
T1–T2	50	35	15	0.739
T3–T4	14	11	3	
Lymph node				
N0	40	25	15	0.044
N1-2	24	21	3	
TNM stage (AJCC)				
I	35	21	14	0.026
II-III	29	25	4	

Next, we consulted the Cancer Cell Line Encyclopedia (http://www.broadinstitute.org/ccle) database to show the level of PARK2 mRNA expression in H1299, HCC827, H1975, and H460 cell lines. The results showed that the lowest expression of PARK2 mRNA was observed in H1299 cells, and the highest expression was observed in H460 cells. Additionally, the H460 cell line (human large cell lung cancer cell line) and the H1299 cell line are human NSCLC cell lines, which are representative and suitable cell lines for this study.

### Overexpression and Knockdown of PARK2 Are Observed in NSCLC Cells

A *PARK2* overexpression lentivirus vector and a negative control lentivirus vector were stably transfected into H1299 cells (H1299-PARK2 vs. H1299-NC) and H460 cells (H460-PARK2 vs. H460-NC), respectively. A small hairpin RNA-PARK2 (sh-PARK2) lentivirus vector and a sh-negative control lentivirus vector were stably transfected into the H460 cells (H460-shPARK2 vs. H460-shNC). The transfection results showed that the level of PARK2 mRNA and protein in H1299-PARK2 and H460-PARK2 cells were significantly increased after overexpression. The level of PARK2 mRNA and protein in H460-shPARK2 cells were obviously decreased after knockdown compared with their control group (*P* < 0.05), as detected by quantitative real-time-polymerase chain reaction (qRT-PCR) and Western blot (Figures 1C,D). The immunofluorescence assay further confirmed that PARK2 was located in the nucleus and cytoplasm, and the level of expression significantly increased or decreased after PARK2 overexpression or knockdown (Figure 1E). Then, the stable transfection cells were used to investigate the potential role of PARK2 in NSCLC.

### Overexpression of PARK2 Suppresses NSCLC Cell Proliferation and Colony Formation

**Figure 2:**
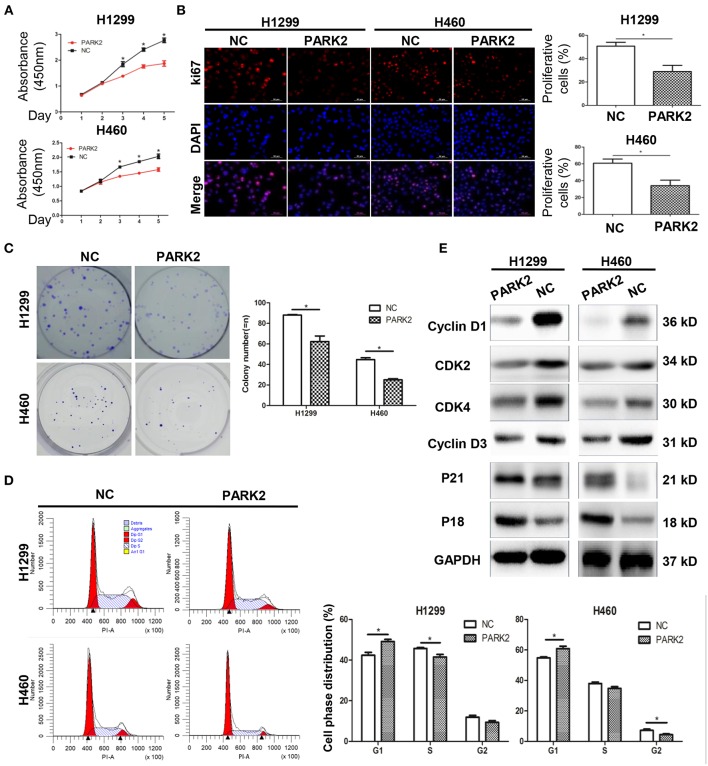
Overexpression of PARK2 suppresses proliferation, colony formation, and cell cycle progression in NSCLC cell lines, H1299 and H460. **(A)** Overexpression of PARK2 significantly inhibits cell viability ability. **(B)** PARK2 reduced cell proliferation as shown by Ki-67 immunofluorescence. **(C)** PARK2 significantly suppresses colony formation. **(D)** Flow cytometry analysis: PARK2 arrested cell cycle at the G1–S transition. (x-axis, DNA content; y-axis, number of cells) **(E)** Western blot assay: altered cell cycle-related proteins altered by PARK2 *P < 0.05.

Compared with control cells, overexpression of *PARK2* in H1299 and H460 cells significantly suppressed cell growth (*P* < 0.05; Figure 2A) and the potential of colony formation (*P* < 0.05; Figure 2B). Consistently, Ki-67 staining showed that the overexpression of *PARK2* led to fewer proliferating cells compared with control cells in H1299 and H460 cell lines (H1299: 28.99 ± 5.29 vs. 50.74 ± 3.26%; H460: 34.17 ± 6.48 vs. 60.8 ± 4.89%; *P* < 0.05; Figure 2C).

### Overexpression of PARK2 Inhibits the NSCLC Cell Cycle

Exploration of the suppressive effects of PARK2 on cell growth by flow cytometry assay showed that the overexpression of *PARK2* significantly arrested cell cycle distribution. H1299-PARK2 increased the percentage of G1 phase cells and decreased the percentage of S phase cells compared with H1299-NC (G1 phase: 49.14 ± 1.05 vs. 43.37 ± 1.41%; S phase: 41.51 ± 1.28 vs. 45.73 ± 0.51%; *P* < 0.05; Figure 2D). H460-PARK2 increased the percentage of G1 phase cells and decreased the percentage of G2 phase cells compared with H460-NC (G1 phase: 60.82 ± 1.64 vs. 54.77 ± 0.66%; G2 phase: 4.41 ± 0.56 vs. 7.24 ± 0.82%; *P* < 0.05; Figure 2D). Consistently, PARK2 regulated the expression of several key proteins in the cell cycle, which was verified by Western blot. G1, S, and G2 phase cell cycle arrest was confirmed by the reduced expression of cell cycle progression regulators, such as cyclin D1, CDK4, cyclin D3, and CDK2, and the elevated expression of cell cycle inhibitors, such as P18 and P21 (Figure 2E).

### Overexpression of PARK2 Induces NSCLC Cell Apoptosis

**Figure 3:**
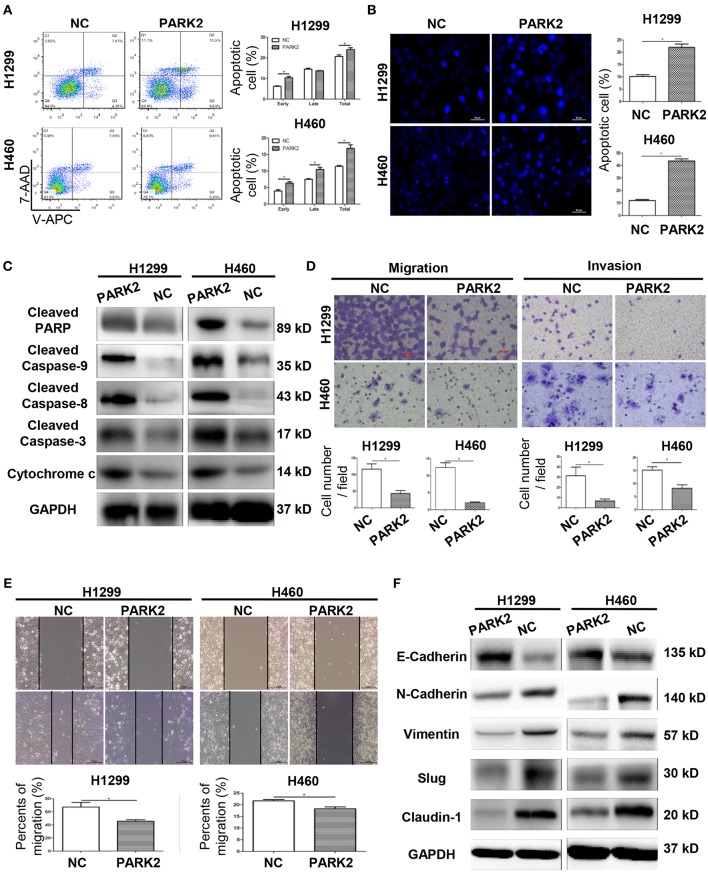
Overexpression of *PARK2* induces cell apoptosis and inhibits the migration and invasion of H1299 and H460 cells. **(A)** After annexin V-APC and 7-aminoactinomycin (7-AAD) double staining by flow cytometry analysis, cell apoptosis assay illuminated that Q2 showed the late apoptotic cells and Q3 showed the early apoptotic cells. **(B)** Cell apoptosis was confirmed by Hoechst 33342. **(C)** Upregulation of apoptosis-related proteins in *PARK2* overexpression cells was verified by western blot assay. **(D)** Overexpression of *PARK2* inhibited cell migration ability and cell invasive ability (magnification × 400). **(E)** In wound healing assay, the results confirmed that PARK2 decreased the migration ability of lung cancer cells. **(F)** PARK2 caused a decrease in cell migration and invasion and regulated of the expression of key EMT proteins as verified by western blot assay *P < 0.05.

Flow cytometry verified that the overexpression of *PARK2* induced a significant increase in total apoptotic cells compared to negative control (H1299: 24.05 ± 0.89 vs. 20.72 ± 0.74%; H460: 16.86 ± 1.07 vs. 11.45 ± 0.23%; *P* < 0.05; Figure 3A). Compared to negative control groups, more apoptotic cells were stained with Hoechst 33342 in the *PARK2* overexpression groups (*P* < 0.05; Figure 3B). Furthermore, the overexpression of *PARK2* increased the levels of protein cytochrome c, caspase-3, caspase-8, and caspase-9 and cleaved PARP expression in H1299 and H460, as detected by Western blot (Figure 3C).

### Overexpression of PARK2 Inhibits the Migration and Invasion of NSCLC Cells

In H1299 and H460 cell lines, the overexpression of *PARK2* obviously suppressed the migration and invasion capabilities of NSCLC cells. The results of a transwell migration assay confirmed that *PARK2* overexpression is associated with decreased in cell migration (*P* < 0.05; Figure 3D). A wound healing assay further confirmed that PARK2 decreased cell recolonization into the wound area compared with control groups (*P* < 0.05; Figure 3E). The transwell invasion assay verified that PARK2 significantly weakened the invasive cells of the H1299 and H460 cell lines compared with that in control group (H1299: 6.74 ± 1.1 vs. 31.37 ± 4.86 cells per field; H460: 8.2 ± 1.35 vs. 15.21 ± 1.29 cells per field; *P* < 0.05; Figure 3D).

Protein expression of epithelial–mesenchymal transition (EMT) markers was detected in H1299 and H460 cell lines by Western blot analysis to explore the mechanism underlying the suppression of cell migration and invasion. As shown in Figure 3F, the overexpression of *PARK2* increased the protein expression of E-cadherin and decreased that of N-cadherin, vimentin, slug, and claudin-1 in the H1299-PARK2 and H460-PARK2 cell lines vs. control groups.

### Knockdown of PARK2 Enhances Cell Viability, Increases Cell Cycle Progression, Promotes Cell Migration and Invasion, and Reduces Cell Apoptosis *in vitro*

**Figure 4:**
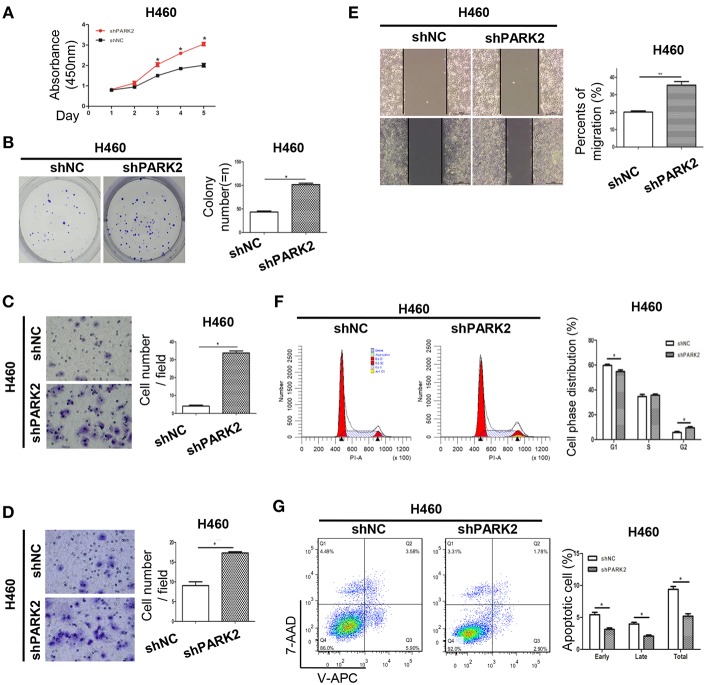
Knockdown of *PARK2* enhances cell viability, increases cell cycle progression, and reduces cell apoptosis in H460 cells. **(A–G)** Knockdown of *PARK2* significantly increased cell viability **(A)**, colony formation **(B)**, migration **(C)**, invasion ability **(D)**, wound healing **(E)**, and cell cycle progression (x-axis, DNA content; y-axis, number of cells) **(F)**, but reduced cell apoptosis **(G)** *P < 0.05.

Knockdown of *PARK2* significantly increased cell growth and colony formation compared with negative control groups (Figures 4A,B). In the transwell migration and wound healing assays, knockdown of *PARK2* promoted cell migration (Figures 4C,E). Invasion of the H460 cell line was enhanced by knockdown of *PARK2* in the transwell invasion assay (Figure 4D). Knockdown of *PARK2* reduced cells in the G1 phase and increased cells in the G2 phase (Figure 4F), further decreasing cell apoptosis (Figure 4G). Accordingly, the tumor suppressive role of PARK2 was further supported.

### Overexpression of PARK2 Inhibits Tumor Growth in a Murine Subcutaneous Xenograft Model

**Figure 5:**
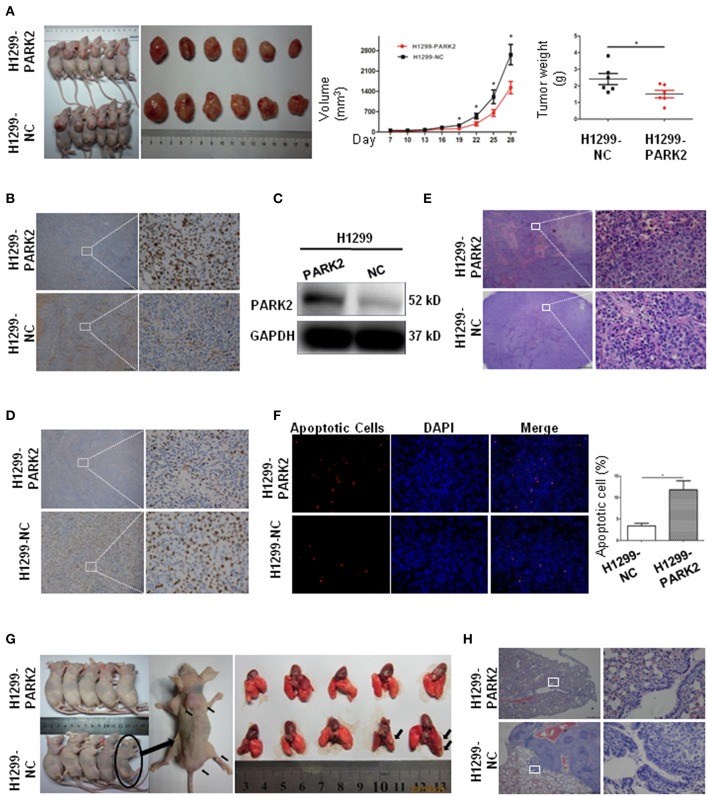
Subcutaneous xenografts and metastasis confirmed the suppressive effect of PARK2 on *in vivo*. **(A)** The image of tumor growth in nude mice subcutaneously injected with H1299-PARK2 and H1299-NC cells. Tumor volumes and weight were compared. **(B–C)** PARK2 protein levels in a murine subcutaneous xenograft model, as confirmed by IHC staining (left panels: magnification × 40, right panels: magnification × 400) and western blot assay. **(D)** As measured by IHC Ki-67, fewer proliferating cells were evaluated in PARK2-expressing subcutaneous xenografts (left panels: magnification × 40, right panels: magnification × 400). **(E)** More cell necrosis was confirmed by H&E staining of H1299-PARK2 tumor nodules in a subcutaneous xenograft model (left panels: magnification × 40, right panels: magnification × 400). **(F)** As detected by TUNEL staining, more apoptotic cells were evaluated in the H1299-PARK2 subcutaneous xenografts. **(G)** Multiple metastases in the muscles and sacrum were significantly detected in the H1299-NC group of metastatic models. The metastasis rate was compared at the end of the experiment. Metastases in the lung were significantly detected in the H1299-NC group of metastatic models. **(H)** The lung transfer xenograft model was verified by H&E staining. *P < 0.05.

A subcutaneous BALB/c^nu/nu^ nude mouse xenograft model with H1299-PARK2 and H1299-NC cells was established by subcutaneous injection (*n* = 12) to explore the effect of PARK2 on tumor growth *in vivo* (Figure 5A). Tumor volumes and body weight of mice were measured every 3 days. After 4 weeks, the tumor growth was significantly delayed in the H1299-PARK2 group relative to the H1299-NC group, and the tumor volume and tumor weight of the two groups were 1531 ± 211.8 vs. 2665 ± 351.0 mm^3^ and 1.51 ± 0.23 vs. 2.41 ± 0.33 g (*P* < 0.05; Figure 5A). Higher expression of *PARK2* in xenograft tumors of the H1299-PARK2 group than that of the H1299-NC group was confirmed by IHC and Western blot (Figures 5B,C). Consistently, fewer positive cells in *PARK2* overexpression subcutaneous xenografts were detected by IHC Ki-67 assays (Figure 5D). Hematoxylin and eosin (H&E) staining confirmed the presence of more necrosis in the tumor nodules of the H1299-PARK2 xenograft than in the H1299-NC xenograft (Figure 5E). Figure 5F showed that the overexpression of *PARK2* induced more cell apoptosis in the H1299-PARK2 group than that in the control group, as detected by TUNEL staining (*P* < 0.05).

### PARK2 Attenuates the Occurrence of Metastasis in Mice

The stable transfection of H1299-PARK2 and H1299-NC cells were employed to construct a metastatic model through tail vein injection of nude mice (Figure 5G). After 6 weeks, no visible tumor nodes were observed in the H1299-PARK2 group. However, two mice in the H1299-NC group had visible tumor nodules on their lung surfaces (Figure 5G). Multiple metastases were also observed in subcutaneous tissue, muscles and sacrum (Figure 5G). Tumor sections were subjected to H&E staining (Figure 5H). In summary, the results strongly suggested that PARK2 significantly inhibited tumor progression and metastasis *in vivo*.

### PARK2 Inhibits the EGFR/AKT/Mammalian Target of Rapamycin (mTOR) Signaling Pathway

**Figure 6:**
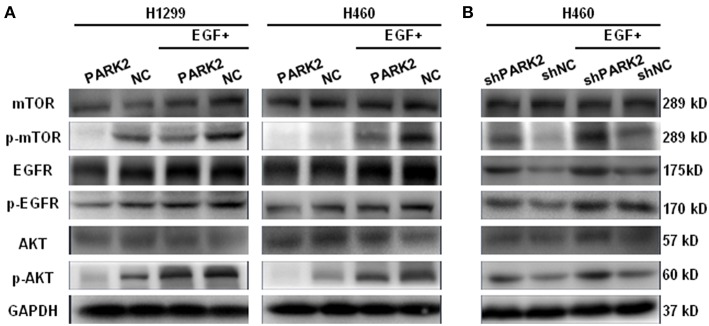
PARK2 inhibits EGFR/AKT/mTOR signaling. **(A)** Western blot analysis showed that overexpression of *PARK2* could suppress EGFR and other phosphorylated targets and mitigate EGF-stimulated conditions (50 ng/mL EGF for 5 min). **(B)** Conversely, knockdown of *PARK2* promoted the pathway. However, AKT and mTOR were not altered. (The proteins were run on different gels).

Western blot assay showed that EGFR was downregulated by the overexpression of *PARK2* in H1299 and H460 cells (Figure 6A). Meanwhile, p-EGFR, p-AKT, and p-mTOR were clearly suppressed by PARK2 (Figure 6A). The effect of PARK2 under EGF stimulation (50 ng/mL EGF for 5 min) was investigated to confirm these results. Moreover, the overexpression of *PARK2* significantly mitigated the EGF-induced protein expression of p-EGFR, p-AKT, and p-mTOR. Comparatively, knockdown of *PARK2* elevated the levels of these phosphorylated proteins (Figure 6B). AKT and mTOR were unaltered following *PARK2* overexpression or knockdown.

## Discussion

*PARK2* alterations were originally recognized as a causal factor for Parkinson's disease (9). The study subsequently showed that *PARK2* was located in the common fragile site (CFS) region in FRA6E as a tumor suppressor. However, the CFS region frequently caused genomic instability by deletions and alterations, particularly in cancer cells (28). Previous studies reported that mutation or loss of *PARK2* might be associated with tumorigenicity by regulating cell growth, cell cycle and mitochondrial biogenesis (29–31). Although several studies suggested that PARK2 is associated with lung cancer, the clear and comprehensive biological functions of PARK2 in NSCLC remain unclear (23, 32).

In this study, we observed that the expression of PARK2 was reduced in human NSCLC tissues compared with their adjacent non-neoplastic tissues by Western blot and IHC staining assays, which are consistent with study in colorectal cancer (33). PARK2 expression was associated with advanced TNM stage, lymph node metastasis and histological grade. Also, the expression of PARK2 between adenocarcinoma and squamous carcinoma have not statistically significant difference. Furthermore, PARK2 was aberrantly expressed in NSCLC cell lines. This study elucidated the biological functions and the underlying molecular mechanism of PARK2 in NSCLC *in vitro* and *in vivo*.

PARK2 was assumed to be involved in cell cycle progression (34). Gong et al. reported that PARK2 mediated the degradation of cyclin D1 by the proteasome, as the proteasome inhibitor MG132 could reverse it (29). Inactivation of PARK2 accounts for the abnormal accumulation of cyclin D and cyclin D–CDK4 complex, which eventually accelerates cell cycle progression (29). Cyclin D–CDK4 activity is restrained by P21 and P18, which are potent inhibitors of G1–S cell cycle transition (35). Moreover, CDK2 is the pivotal cell cycle regulator in the S phase and G2 phase (36). In the present study, PARK2 arrested a significant percentage of NSCLC cells at the G1 phase, upregulated the activation of P21 and P18, downregulated the activation of cyclin D1, cyclin D3, and CDK4 and reduced the Ki-67 index. Moreover, the decreased S phase cells were confirmed by the reduction in CDK2 expression. Meanwhile, overexpression of *PARK2* significantly suppressed NSCLC cell proliferation and colony formation. These findings were consistent with the *PARK2* knockdown results, which showed that PARK2 deficiency promoted cell proliferation and colony formation.

As we know, when apoptosis is inhibited and cell cycle progression is uncontrolled, malignant transformation of cells increases. Similarly, our study determined that PARK2 induced more apoptosis in NSCLC cells *in vitro* and increased expression of protein cytochrome c, caspase-3, caspase-8, and caspase-9 and cleaved PARP. Caspase-3 effectors were triggered by activated caspase-8 and caspase-9. Then, the proteolytic cleavage of PARP was further triggered during the apoptotic process (37–39). Moreover, the subcutaneous xenograft model illustrated that *PARK2* highly attenuated tumorigenesis and development of NSCLC in nude mice. In addition, further pathological analysis showed that significantly more proliferative cells and less apoptotic cells were detected in the subcutaneous xenograft tumors of the negative control group. Consistent with previous findings (40), PARK2 suppressed NSCLC cell proliferation and induced apoptosis.

Next, we explored the mechanisms underlying PARK2-inhibited metastasis. The data showed that overexpression of *PARK2* significantly suppressed migration and invasion of NSCLC cells *in vitro* and dramatically reduced metastatic nodules *in vivo*. EMT contributes pathologically to cancer progression by promoting mesenchymal adhesion. In particular, the downregulation of E-cadherin and the upregulation of N-cadherin altered cell adhesion and contributed to tumor metastasis (41–43). In the present study, we found that PARK2 reduced invasion by regulating several important members of the EMT, including upregulating E-cadherin and downregulating N-cadherin, vimentin, slug, and claudin-1.

The PI3K/AKT/mTOR signaling pathway plays a vital role in processes related to cell growth, proliferation, metabolism, migration, and survival. Abnormal activation of the pathway is linked to diseases and tumorigenesis (44–48). Lin et al. identified that EGFR and its downstream pathway were potently suppressed by ubiquitination of PARK2 in glioma. Therefore, dysfunction of PARK2 led to the deficiency of E3 ligase and upregulation of the EGFR/AKT pathway (49). Using a similar approach, Yeo et al. (50) determined that the overexpression of *PARK2* downregulated AKT phosphorylation in U87 cells. A recent report showed that the depletion of PARK2 enhanced the activation of PI3K/AKT/mTOR and played an important role in maintaining the activation of PENT, which was a well-known tumor suppressor that inhibits the PI3K/AKT pathway (51). Consistent with previous findings, our study confirmed that PARK2 exerted a tumor suppressive effect by regulating the activation of EGFR in NSCLC and inhibiting p-EGFR, p-AKT, and p-mTOR. Thus, overexperssion of *PARK2* in NSCLC cells reduced the level of cyclin D1 and N-cadherin and enhanced expression of caspase-3 and E-cadherin. Ultimately, PARK2 regulated the expression of those critical proteins by altering the activity of EGFR, AKT, and mTOR. These findings elucidated the underlying mechanisms of the tumor suppressive property of PARK2 in NSCLC, which inhibited the EGFR/AKT/mTOR pathway to suppress the growth, metastasis and promotes apoptosis of NSCLC cells. Moreover, these findings indicated that *PARK2* acts as a tumor suppressor gene and negatively regulates tumorigenesis through multiple pathways.

## Conclusion

In summary, we have identified that *PARK2* acted as a tumor suppressor gene that negatively regulates tumorigenesis and progression in NSCLC. PARK2 exerts a tumor suppressive function *in vitro* and *in vivo*, including retarding growth, migration, invasion, and metastasis and promotes apoptosis of NSCLC cells through inhibition of the EGFR/AKT/mTOR pathway. These results improve our understanding of NSCLC progression and supports PARK2 as a novel biomarker for NSCLC.

## Data Availability

The raw data supporting the conclusions of this manuscript will be made available by the authors, without undue reservation, to any qualified researcher.

## Ethics Statement

This research was approved by the Ethics Committee of the second Affiliated Hospital, School of Medicine, Zhejiang University, which is accredited by the National Council on Ethics in Human and Animal Research.

## Author Contributions

SZ and CP conceived the study and provided suggestions and supervision of the study. HD and ZL conducted experiments, acquired and analyzed data, and wrote the manuscript. FX and PD participated in experiments. TP and DL contributed to the tissue specimens. CZ contributed to data analysis. All authors corrected draft versions and approved the final version of the manuscript.

### Conflict of Interest Statement

The authors declare that the research was conducted in the absence of any commercial or financial relationships that could be construed as a potential conflict of interest.
